# Tamoxifen attenuates manganese-induced dysregulation of neuronal REST via the genomic ER-*α* mechanism

**DOI:** 10.3389/fnmol.2025.1648904

**Published:** 2025-09-15

**Authors:** Alexis Digman, Edward Pajarillo, Sanghoon Kim, Itunu Ajayi, Deok-Soo Son, Michael Aschner, Eunsook Lee

**Affiliations:** ^1^Department of Pharmaceutical Sciences, Florida A&M University, Tallahassee, FL, United States; ^2^Department of Biochemistry, Cancer Biology, Neuroscience and Pharmacology, Meharry Medical College, Nashville, TN, United States; ^3^Department of Molecular Pharmacology, Albert Einstein College of Medicine, Bronx, NY, United States

**Keywords:** tamoxifen, nuclear ER-*α*, manganese, NRSF/REST, SERM

## Abstract

Chronic exposure to elevated levels of manganese (Mn) causes a neurological disorder referred to as manganism, resembling pathological symptoms of Parkinson’s disease (PD). The repressor element-1 silencing transcription factor (REST) induces neuroprotection in several neurological disorders, including PD and Mn toxicity. Tamoxifen (TX), a selective estrogen receptor modulator, has been shown to afford neuroprotective effects in various experimental models and increase REST expression via the non-genomic estrogen receptor (ER)/Wnt signaling in Cath. a-differentiated (CAD) neuronal cultures. The present study investigated whether TX enhances REST transcription through the genomic estrogen receptor (ER) pathway in CAD cells, using a combination of Western blotting, quantitative reverse transcription polymerase chain reaction (qRT-PCR), promoter activity assays, chromatin immunoprecipitation, electrophoretic mobility shift assays, and site-directed mutagenesis. The findings showed that the REST promoter sequences contained half-site estrogen response elements (ERE) motifs. The ER-*α* pathway primarily upregulated REST, as the ER-α selective agonist propylpyrazole triol (PPT) (1 μM) predominantly increased REST transcription and attenuated Mn (250 μM)-induced REST reduction in CAD cells. TX induced REST upregulation by activation of the genomic ER-*α* pathway, as it increased nuclear ER-*α*’s interaction with cyclic adenosine monophosphate (AMP) response element (CREB)-binding protein and Sp1 and promoted ER-α binding to the half-site ERE in the REST promoter. Moreover, the ERE mutation in the REST promoter reduced TX-induced REST promoter activity, and TX reversed Mn-induced REST transcriptional repression. Our novel findings suggest that the genomic ER-*α* pathway plays a critical role in TX-induced REST upregulation and mitigation of Mn-induced decreases in REST expression.

## 1 Introduction

Chronic exposure to elevated levels of manganese (Mn) induces a neurological disorder known as manganism (Wennberg et al., 1991), which shares similar symptoms with Parkinson’s disease (PD), such as tremor, rigidity, shuffling gait, and cognitive dysfunction (Lucchini et al., 2009; Kornblith et al., 2018). Humans are exposed to excessive Mn levels in occupational settings such as welding and mining (Rodier, 1955; Wang et al., 1989), as well as in environmental sources, including contaminated air and drinking water (Kornblith et al., 2018; Aiken and Ying, 2023). Upon ingestion, Mn preferentially accumulates in the basal ganglia (Newland et al., 1989), leading to impairment of dopaminergic neurons in the nigrostriatal pathway, which is responsible for the motor function deficits. At the cellular and molecular levels, Mn induces dopaminergic toxicity through several mechanisms, including mitochondrial impairment, oxidative stress, inflammation, apoptosis, and dysregulation of gene expression (Harischandra et al., 2019). In addition, Mn disrupts the dopaminergic system by decreasing the expression and activation of tyrosine hydroxylase (TH), the rate-limiting enzyme in dopamine synthesis, leading to reduced dopamine levels (Zhang et al., 2011; Kumasaka et al., 2017; Pajarillo et al., 2020). Our previous studies have shown that Mn decreased TH expression by dysregulating transcription factor (TF) neuron-restrictive silencing factor/repressor element-1 silencing transcription factor (NRSF/REST), which positively regulated TH transcription by binding to the REST binding motifs in the TH promoter (Pajarillo et al., 2020). Accordingly, investigating the pharmacological intervention involved in REST upregulation is critical for the development of new therapeutic strategies to alleviate Mn-induced dopaminergic toxicity.

REST has drawn attention for its protective effects in several clinical and experimental models of neurological disorders, such as Alzheimer’s disease (AD) (Lu et al., 2014; Ashton et al., 2017), PD (Yu et al., 2013; Kawamura et al., 2019), and Mn toxicity (Pajarillo et al., 2020; Pajarillo et al., 2021; Pajarillo et al., 2024). REST is an essential TF involved in regulating cellular functions such as neurogenesis, differentiation, axonal growth, and vesicular transport (Schoenherr and Anderson, 1995; Paquette et al., 2000; Ballas et al., 2005). REST was originally discovered as a repressor of neuronal genes in non-neuronal cells (Chen et al., 1998; Jones and Meech, 1999), but several studies have reported that it also serves as an activator, offering neuroprotective effects against multiple neurological disorders (Lu et al., 2014; Pajarillo et al., 2020; Pajarillo et al., 2021). In AD models, REST increased transcription of antioxidant genes, such as superoxide dismutase 1 (SOD-1) and catalase, and antiapoptotic genes, including Bcl-2 (Lu et al., 2014). Furthermore, REST appears to induce neuroprotection against Mn toxicity, supported by the findings that REST mitigated Mn-induced neurotoxicity by increasing antioxidant, anti-apoptotic proteins, as well as decreasing Mn-increased pro-inflammatory cytokines in dopaminergic neuronal cells (Pajarillo et al., 2020). REST also mitigated Mn-induced glutamate excitotoxicity by increasing glutamate transporters in astrocytes (Pajarillo et al., 2021). These findings collectively highlight the role of REST as an activator and a neuroprotective TF.

17β-estradiol (E2), a female sex hormone, has demonstrated neuroprotective effects in several experimental models of neurodegenerative diseases (Cui et al., 2013, for review) as well as in Mn toxicity (Lee et al., 2009a; Lee et al., 2009b; Lee et al., 2013). E2 has been shown to exert neuroprotection via the nuclear estrogen receptor (ER)-mediated genomic pathway, which directly regulates transcription of target genes (Brann et al., 2012, for review). However, the use of E2 as a neuroprotectant is limited by its peripheral unwanted effects; thus, it is crucial to develop E2-like compounds that exert neuroprotection with an improved safety profile, exerting their protective effects only in the brain. Several selective estrogen receptor modulators (SERMs), such as tamoxifen (TX) (Dhandapani and Brann, 2003; Zhang et al., 2007; Lim et al., 2018; Finney et al., 2021) and raloxifene (Ciriza et al., 2004; Poirier et al., 2016; Nohara et al., 2023), have shown promise in eliciting neuroprotective effects in neurotoxicity models. For example, TX exerted neuroprotection in ischemia (Zhang et al., 2005), hippocampal silent infarcts (Zou et al., 2015; Finney et al., 2021), and psychiatric disorders (Novick et al., 2020). TX also protected against Mn-induced neurotoxicity in rat primary neurons and astrocytes, as well as in an *in vivo* mouse model (Lee et al., 2009a; Lee et al., 2009b; Karki et al., 2014; Pajarillo et al., 2018). Recently, we reported that TX increased REST expression via ER-*α*/Wnt/*β*-catenin signaling in dopaminergic neuronal cells, leading to protection against Mn-induced neurotoxicity (Digman et al., 2025). For the ER mechanisms, ER-*α* has been shown to mediate TX-induced neuroprotection in the traumatic brain injury rat model (Lim et al., 2018), highlighting its role in mediating TX-induced neuroprotection.

Given the above, in the present study, we investigated whether TX increases REST transcription and mitigates Mn-induced REST downregulation via the genomic ER mechanism in Cath. a-differentiated (CAD) neuronal cultures. Our findings demonstrate that the REST promoter contains the half-site ERE, and that TX upregulates REST transcription by activating the genomic ER-α pathway, which involves increasing ER-α binding to this ERE in the REST promoter. TX also alleviated the Mn-induced decrease in REST transcription via the same ER-α-mediated mechanism in CAD cells.

## 2 Materials and methods

### 2.1 Materials

Manganese (II) chloride (MnCl₂), TX, REST antibody (07–579), and dimethyl sulfoxide (DMSO) were obtained from MilliporeSigma (Burlington, MA, USA). The selective ER agonists propylpyrazole triol (PPT) and diarylpropionitrile (DPN) were purchased from BioTechne, Tocris (Minneapolis, MN, USA). Cell culture reagents, including trypsin-ethylenediaminetetraacetic acid (EDTA), Minimum Essential Medium (MEM), Dulbecco’s Modified Eagle Medium/Nutrient Mixture F-12 (DMEM/F-12), and cell culture components were sourced from Thermo Fisher Scientific Inc. (Waltham, MA, USA). Antibodies against ER-α (sc-543), cyclic adenosine monophosphate (AMP) response element (CREB)-binding protein (CBP)/p300 (sc-32244), Sp1 (sc-17824), and *β*-actin (sc-47778) were obtained from Santa Cruz Biotechnology (Santa Cruz, CA, USA). Histone H3 (ab1791) and secondary antibodies, including Horseradish Peroxidase (HRP)-conjugated rabbit anti-mouse IgG (ab6728), HRP-conjugated goat anti-rabbit IgG (ab6721), and Alexa Fluor 488-conjugated goat anti-mouse IgG (ab150113), were purchased from Abcam (Cambridge, MA, USA). All chemicals were dissolved in phosphate-buffered saline (PBS), double-distilled water, or DMSO, and diluted to final working concentrations in Opti-MEM prior to application. The human REST promoter plasmid was kindly provided by Dr. Yvon Trottier (INSERM, France).

### 2.2 Cell culture

The catecholaminergic mouse CAD cell line (catalog no. 08100805) was obtained from MilliporeSigma. Cells were cultured in DMEM/F-12 medium supplemented with 2 mM L-glutamine, 10% fetal bovine serum (FBS), 1 × GlutaMAX™, 100 U/mL penicillin, and 100 μg/mL streptomycin. Neuronal differentiation was induced by switching to serum-free medium. Cells were dissociated using 0.125% trypsin and 0.1 g/L EDTA, then seeded into 6-well or 24-well plates for functional assays, or into 100 mm × 20 mm or 150 mm × 20 mm culture dishes for promoter activity, RNA, or protein analyses. Cultures were maintained at 37°C in a humidified incubator with 5% CO₂ and 95% air.

### 2.3 Transient transfections

Cells were transfected with plasmid DNA using Lipofectamine™ 3,000 (Thermo Fisher Scientific) according to the manufacturer’s instructions. Plasmid vectors were introduced at a concentration of 0.1–1.0 μg per 5.0 × 10^5^ cells. Following transfection, cells were left for at least one night before subsequent assays and analyses were performed.

### 2.4 Measurement of promoter activity

CAD cells were transfected with the human REST promoter plasmid vectors, followed by overnight incubation. Transfected cells were treated with various compounds, and REST promoter activity was measured using the Bright-Glo™ Luciferase Assay System (Promega, Madison, WI) according to the manufacturer’s instructions.

### 2.5 Western blot

Following treatment with the indicated compounds, CAD cells were washed with ice-cold PBS and lysed in radioimmunoprecipitation assay (RIPA) buffer supplemented with a protease inhibitor cocktail. Lysates were collected, and equal amounts of protein were separated on 10% sodium dodecyl-sulfate polyacrylamide gel electrophoresis (SDS-PAGE) gels for subsequent Western blot analysis. Primary antibodies were applied at a 1:1,000 dilution, while HRP-conjugated secondary antibodies were used at dilutions ranging from 1:2,500 to 1:5,000. Protein bands were visualized using the West Pico PLUS chemiluminescence substrate (Pierce, Rockford, IL, USA), and signal detection and quantification were performed using the Bio-Rad ChemiDoc Imaging System and ImageJ software (Bethesda, MD, USA) as described previously (Pajarillo et al., 2020).

### 2.6 Immunocytochemistry (ICC)

CAD cells were cultured on poly-L-lysine–coated glass coverslips in 6-well plates for immunostaining as previously described (Pajarillo et al., 2020). Primary antibodies targeting ER-*α* and CBP/p300 were applied at a 1:250 dilution, followed by incubation with Alexa Fluor® 488-conjugated secondary antibodies (1:1000 dilution). Fluorescence intensity and subcellular localization were analyzed using a Leica SPEII confocal microscope (Leica Microsystems, Inc.). Colocalization analysis of ER-*α* and DAPI fluorescence was performed using ImageJ. Individual nuclei were first identified based on DAPI staining, and three representative nuclei per group were randomly selected as regions of interest (ROIs). Fluorescence intensity profiles for ER-α and DAPI were extracted from each ROI. After treatment with TX across different time points, Pearson’s correlation coefficients were calculated using GraphPad Prism to assess the colocalization overlap between ER-α and the nuclear signal DAPI in a time-dependent manner, while treatment-dependent differences were compared at a single time point.

### 2.7 Nuclear fractionation and co-immunoprecipitation (co-IP)

Cells were lysed in a fractionation buffer composed of 20 mM HEPES (pH 7.4), 10 mM KCl, 2 mM MgCl₂, 1 mM EDTA, 1 mM EGTA, and 1 mM DTT, supplemented with a protease inhibitor cocktail. Mechanical disruption was performed using a syringe, followed by centrifugation at 720 *g* for 5 min at 4°C to separate the cytoplasmic components (supernatant). The nuclear pellet was subsequently resuspended in Tris-buffered saline (TBS) containing 0.1% SDS. Protein concentrations of the nuclear extracts were quantified using the bicinchoninic acid (BCA) assay. For Co-IP, as described previously (Pajarillo et al., 2020), nuclear extracts were incubated and immunoprecipitated with ER-α antibody, followed by Western blotting for CBP/p300 and Sp1.

### 2.8 Quantitative RT-PCR

Following the appropriate treatment, samples from CAD cells were prepared for quantitative polymerase chain reaction (qPCR). To extract total RNA from each group, three samples were processed using the RNeasy Mini Kit (Qiagen, Valencia, CA). Purified RNA (2 μg) was then subjected to reverse transcription using the high-capacity complementary DNA (cDNA) reverse transcription kit (Thermo Fisher Scientific) to generate cDNA. Real-time qPCR was performed on the Bio-Rad CFX96 instrument (Hercules, CA) using the Bio-Rad iQ SYBR Green Supermix and 0.4 μM primers. The reaction volume for each sample was 25 μL, with 1 μL of cDNA template included. The following primers for mouse were utilized: REST forward: 5′-ACT TTG TCC TTA CTC AAG CTC-3′, reverse: 5′-CAT TTA AAT GGC TTC TCA CCT G-3′; GAPDH forward: 5’-CTC ATG ACC ACA GTC CAT GC-3′, reverse: 5’-CAC ATT GGG GGT AGG AAC AC-3′. The qPCR parameters consisted of 1 cycle at 95°C for 10 min, followed by 40 cycles at 95°C for 15 s and 60–65°C for 1 min. mRNA levels were analyzed using Bio-Rad CFX Manager Version 3.1, with GAPDH as the internal control.

### 2.9 Chromatin immunoprecipitation (ChIP) assay

The ChIP assay was conducted using the EZ-ChIP kit from MilliporeSigma, following the manufacturer’s instructions, as previously described (Pajarillo et al., 2020). Real-time qPCR was performed using primers targeting the ERE binding site on the REST promoter (forward: 5′-CCT CTG TCT ACT GAA TTC TGA G-3′, reverse: 5′-CTG GCT GCA CAA GTC TGT AAT C-3′). The immunoprecipitated DNA was quantified by measuring % inputs from qPCR products using the Bio-Rad CFX Manager 3.1 software.

### 2.10 Electrophoretic mobility shift assay (EMSA)

EMSA was conducted using a LightShift chemiluminescent kit from Thermo Fisher Scientific, following the manufacturer’s instructions, as previously described (Pajarillo et al., 2020). The primer pair utilized for targeting the ERE on the REST promoter was as follows: 5′-CCC AAT TTG TCA AGT CAA TGA CCT GGA TCT CCT GG-3′ and 5’-CCA GGA GAT CCA GGT CAT TGA CTT GAC AAA TTG GG-3′. DNA-protein complexes were quantified using ImageLab Software (BioRad).

### 2.11 Site-directed mutagenesis

The half-site ERE binding sequence in the human 5’UTR REST promoter was mutated using the Q5 mutagenesis kit (New England Biolabs, Ipswich, MA, USA) according to the manufacturer’s recommendations. The 5′UTR REST promoter (−3,390/+1) subcloned into the pGL3 basic vector was used as the original template for mutation. The primers used for ERE mutant (ERE-mut) were 5′-GGG CCC CAC TCC CTT TCT-3′ and 5′-GCC TCT TTT CTC AGC TAA GGG CAG G-3′. The ERE mutant clones were confirmed by Primordium sequencing.

### 2.12 Statistical analysis

All data were expressed as the mean ± standard deviation (SD) of the mean. Statistical analyses were performed using either Student’s *t-*test or one-way analysis of variance (ANOVA), followed by *Sidak’s post-hoc* tests using the GraphPad Prism software Version 9.0 (San Diego, CA, USA). A *p*-value of less than 0.05 (*p* < 0.05) was considered statistically significant. The data shown are representative of three independent experiments.

## 3 Results

### 3.1 The REST promoter contains the half-site ERE sequences, and ER-*α* is the predominant ER subtype responsible for upregulating REST expression in CAD neuronal cells

**Figure 1:**
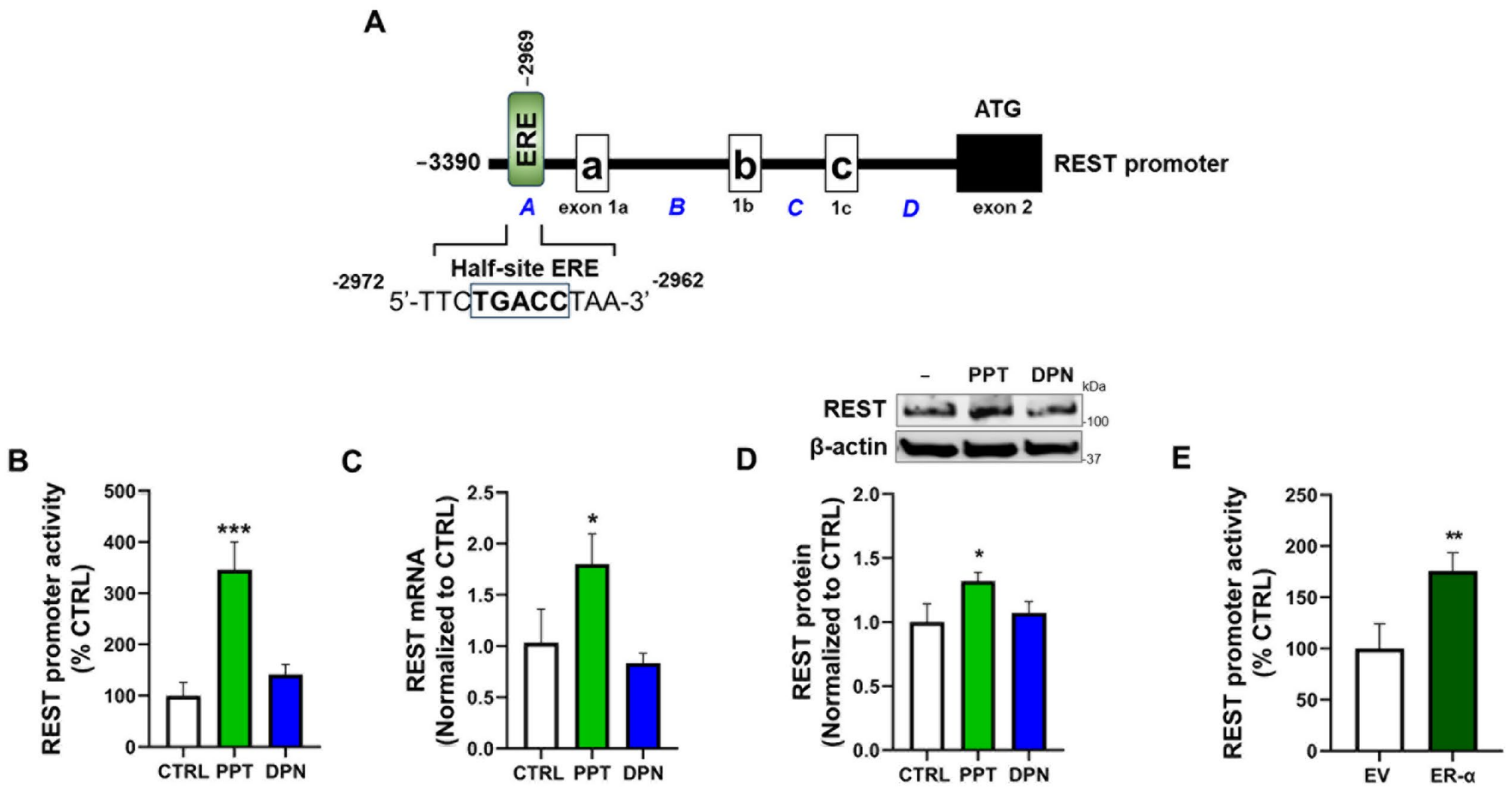
The ER-*α* is the primary ER subtype to increase REST expression in CAD cells. **(A)** Illustration of the human 5′UTR (−3,390/+1) REST promoter region with a half-site ERE (TGACC). **(B)** CAD cells were transfected with a human 5’UTR-REST promoter vector and then exposed to either 1 μM PPT or 1 μM DPN for 6 h, followed by a luciferase assay to measure REST promoter activity. **(C,D)** CAD cells were treated with either 1 μM PPT or 1 μM DPN for 12 h and 24 h, followed by measurement of REST mRNA using qPCR **(C)** and REST protein using Western blot **(D)**, respectively. **(E)** CAD cells were co-transfected with the human REST promoter and ER-α expression vector, followed by a luciferase assay to detect REST promoter activity. GAPDH and β-actin were used as loading controls for mRNA and protein, respectively. Quantification of protein bands was normalized to β-actin. Relative mRNA expression levels were normalized to GAPDH. *p < 0.05, **p < 0.01, and ***p < 0.001, compared to the control. (Student’s *t*-test or one-way ANOVA followed by *Sidak’s post-hoc*, *n* = 3). The data shown are representative of three independent experiments.

The REST promoter sequences have been shown to contain cis-regulatory elements for several TFs, including Sp1 (Ravache et al., 2010) and *β*-catenin/T-cell factor/lymphoid enhancer factor (TCF/LEF) (Digman et al., 2025). Here, we identified a half-site ERE (TGACC) in the REST promoter (Figure 1A), which is known to be capable of activating the transcription of its target genes (Petz and Nardulli, 2000). We also tested if a specific ER subtype modulates REST expression by treating CAD neuronal cultures with subtype-selective ER agonists, PPT (a selective ER-*α* agonist), and DPN (a selective ER-β agonist). The results showed that the ER-α agonist PPT at 1 μM increased REST promoter activity (Figure 1B), as well as mRNA and protein levels (Figures 1C,D), but not 1 μM of DPN, although the lower concentration (100 nM) of DPN moderately increased REST transcription (Supplementary Figure S1). Furthermore, overexpression of ER-*α* increased REST promoter activity (Figure 1E), supporting the critical role of ER-*α* in REST upregulation in CAD cells.

We also investigated whether ER-β modulates ER-α’s effects on REST expression, as ER-β has been shown to negatively regulate ER-*α*’s expression and activity (Hall and McDonnell, 1999). The results showed that co-treatment of DPN with PPT (both 100 nM) decreased PPT’s increasing effects on REST expression (Supplementary Figure S2). Moreover, co-treatment of PHTPP (ER-β antagonist) with PPT further increased PPT effects on REST expression (Supplementary Figure S3), indicating that ER-β negatively regulates the ER-α-REST mechanism.

### 3.2 ER-*α* activation attenuated the Mn-induced decrease in REST transcription via the genomic ER mechanism in CAD cells

**Figure 2:**
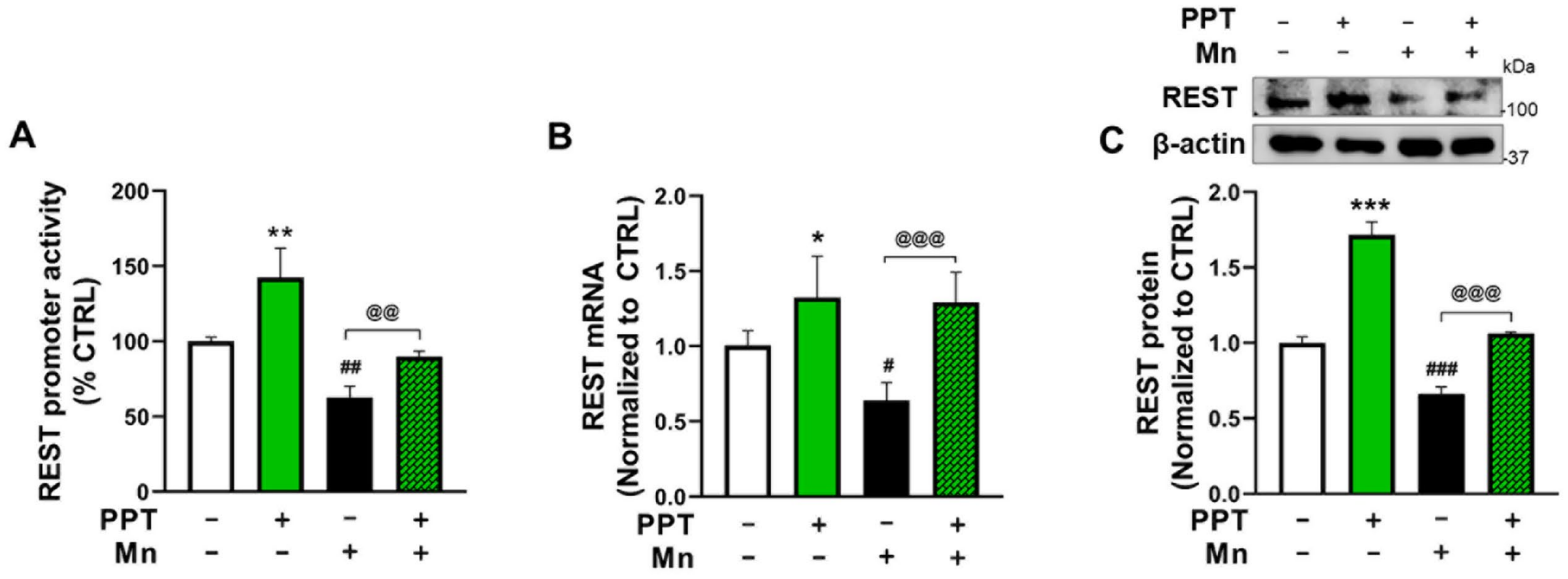
ER-α activation attenuated the Mn-induced decrease in REST transcription in CAD cells. **(A)** CAD cells were transfected with a human 5’UTR-REST promoter vector, then pre-treated with 1 μM PPT for 6 h prior to 250 μM Mn exposure for 6 h (in the presence of PPT), followed by measurement of REST promoter activity. **(B)** CAD cells were pre-treated with 1 μM PPT for 12 h prior to 250 μM Mn exposure for 12 h (in the presence of PPT), followed by measurement of REST mRNA. **(C)** CAD cells were pre-treated with PPT for 24 h, then with 250 μM Mn for another 24 h (in the presence of TX), followed by measurement of REST protein. GAPDH and β-actin were used as loading controls for mRNA and protein, respectively. Quantification of protein bands was normalized to β-actin. Relative mRNA expression levels were normalized to GAPDH. *p < 0.05, **p < 0.01, and ***p < 0.001; ^#^*p* < 0.05, ^##^*p* < 0.01, and ^###^*p* < 0.001, compared to the control. ^@@^*p* < 0.01 and ^@@@^*p* < 0.001, compared to each other. (One-way ANOVA followed by *Sidak’s post-hoc*; *n* = 3). The data shown are representative of three independent experiments.

Since ER-*α* was identified as the primary ER subtype that increases REST transcription, we tested whether ER-α also plays a role in attenuating Mn-induced reductions of REST in CAD neuronal cultures. We have previously shown that Mn decreases REST expression in dopaminergic neuronal cultures (Pajarillo et al., 2020). Our current findings reveal that the ER-*α* selective agonist PPT attenuates Mn-induced decreases in REST promoter activity (Figure 2A), as well as mRNA and protein levels (Figures 2B,C), indicating that ER-α contributes to attenuation of Mn-decreased REST.

**Figure 3:**
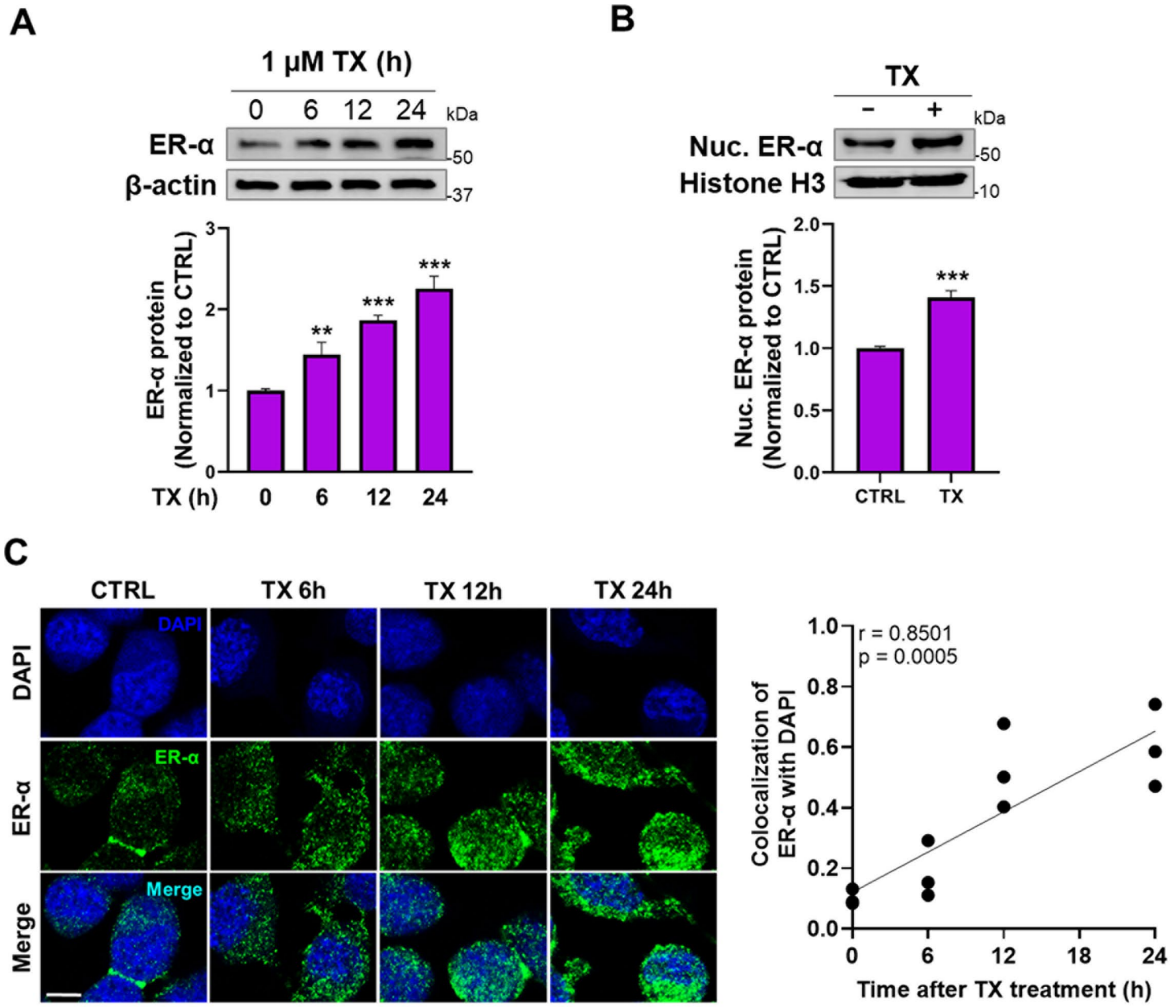
TX enhanced the ER-α expression and its nuclear translocation in CAD cultures. **(A)** CAD cells were treated with 1 μM TX at several time points and detected ER-α protein levels in whole-cell lysates. **(B)** Nuclear ER-α protein levels were detected in nuclear extracts of CAD cells treated with TX for 24 h. **(C)** Immunofluorescence images of ER-α nuclear translocation upon treatment with 1 μM TX for several time points. Scale bar, 20 μM. Pearson’s correlation analysis of quantified nuclear ER-α and DAPI fluorescence signals revealed a strong and statistically significant positive correlation (*r* = 0.8501, *p* = 0.0005), indicating increased nuclear localization of ER-α. β-actin and Histone H3 were used as loading controls of whole-cell lysate and nuclear extracts, respectively. Quantification of protein bands was normalized to β-actin (whole-cell lysate) or histone H3 (nuclear extracts). **p < 0.01 and ***p < 0.001, compared to the control. (Student’s *t*-test or one-way ANOVA followed by *Sidak’s post-hoc*; *n* = 3). The data shown are representative of three independent experiments.

Next, we tested if TX activated the genomic ER-α pathway to attenuate Mn effects, given that the REST promoter sequences contain the half-site ERE motifs. The results showed that TX increased ER-α protein levels in whole-cell lysates (Figure 3A). Furthermore, TX promoted the nuclear translocation of ER-α, as observed by increased nuclear localization in Western blot data (Figure 3B) and immunofluorescence images (Figure 3C). Quantitative analysis of ER-*α* colocalization with the nuclear marker DAPI confirmed a time-dependent increase in nuclear translocation following TX exposure (Figure 3C), consistent with activation of the genomic ER-*α* signaling pathway in our experimental model.

**Figure 4:**
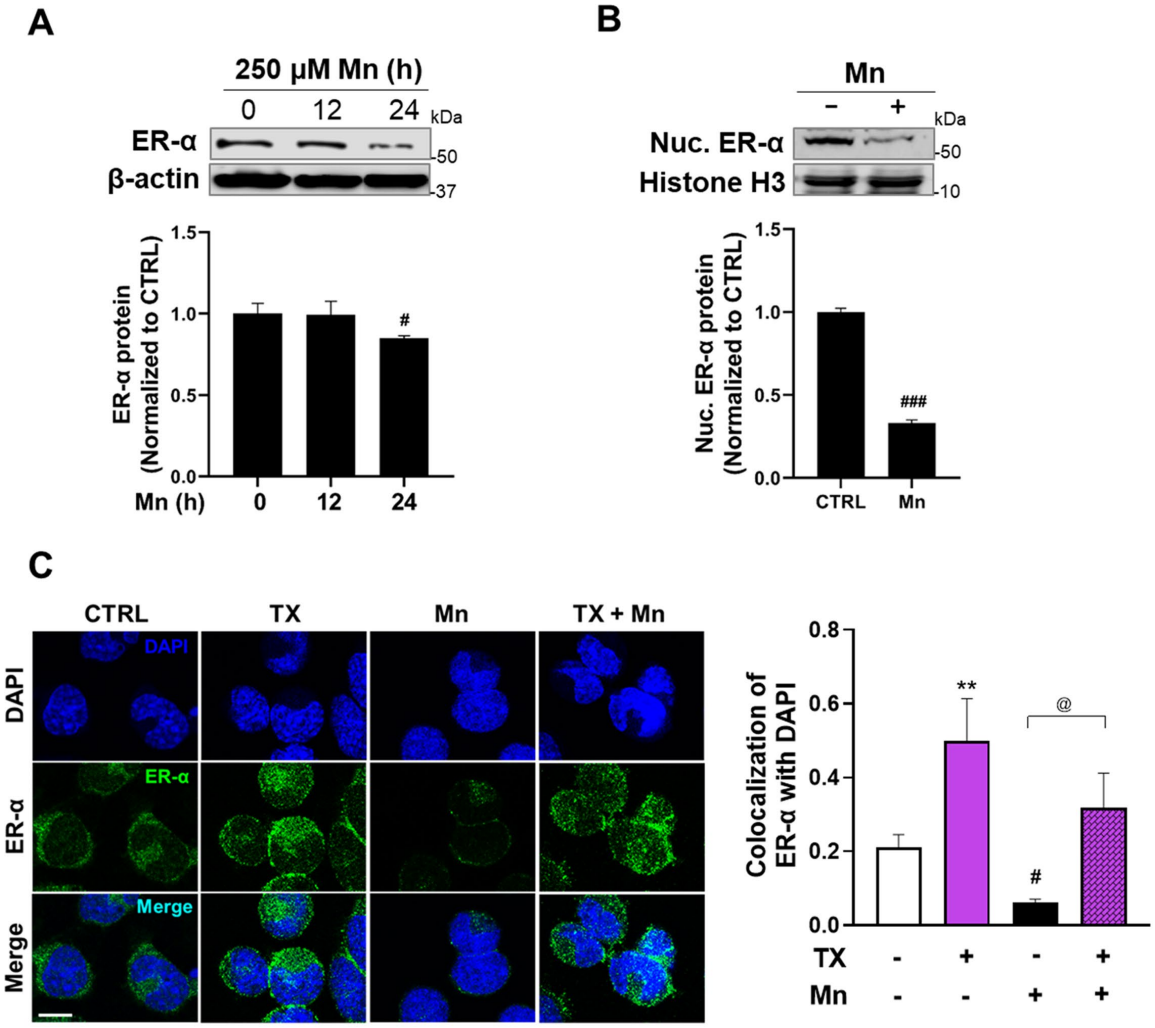
Mn decreased ER-α protein expression, while TX attenuated Mn-induced reductions in ER-α nuclear translocation. **(A)** CAD cells were treated with Mn at several time points, and total ER-α protein levels were detected in whole-cell lysates. **(B)** Nuclear ER-α protein levels were detected in nuclear extracts of CAD cells exposed to Mn for 24 h. **(C)** Immunofluorescence images of ER-α nuclear translocation upon pre-treatment of TX for 12 h, followed by Mn exposure for another 24 h (in the presence of TX) in CAD cells. Comparative analysis of ER-α and DAPI fluorescence colocalization highlights the effects of Mn exposure and TX treatment on ER-α nuclear localization. Scale bar, 20 μM. β-actin and histone H3 were used as loading controls for whole-cell lysate and nuclear extracts, respectively. Quantification of protein bands was normalized to β-actin (whole-cell lysate) or histone H3 (nuclear extracts). **p < 0.01, ^#^*p* < 0.05, and ^###^*p* < 0.001, compared to the control. ^@^*p* < 0.05, compared to each other. (Student’s *t*-test or one-way ANOVA followed by *Sidak’s post-hoc*; n = 3). The data shown are representative of three independent experiments.

On the other hand, Mn decreased total ER-α protein levels (Figure 4A) and its nuclear translocation (Figure 4B), but TX attenuated these Mn-induced inhibitory effects, as shown by the subcellular localization of ER-*α* (Figure 4C). Quantitative analysis of ER-α colocalization with DAPI confirmed that Mn reduced nuclear ER-α, whereas TX attenuated those Mn effects (Figure 4C). These findings indicate that the genomic ER-α pathway plays a critical role in TX’s attenuation of Mn’s inhibitory effects.

### 3.3 TX increased ER-α binding to the ERE in the REST promoter and attenuated Mn effects in the reduction of REST transcription in CAD cells

**Figure 5:**
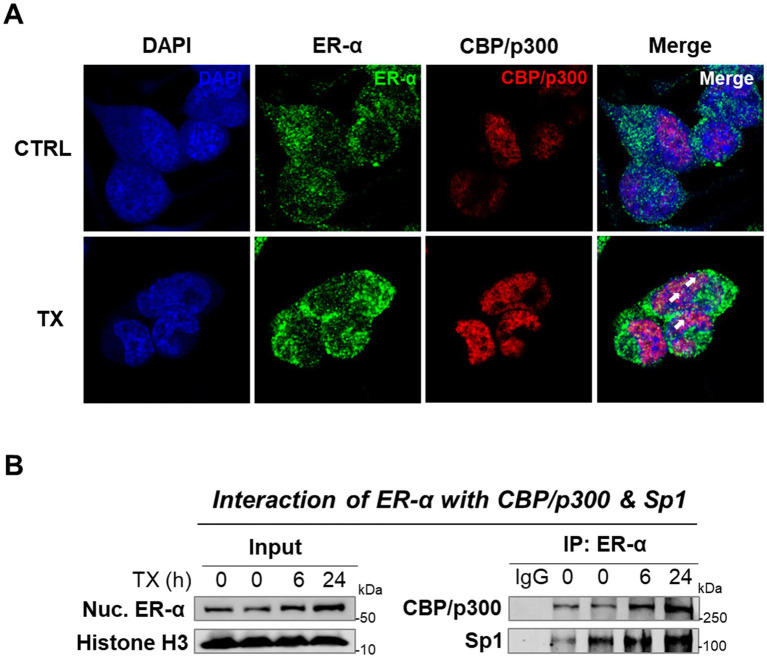
TX increases ER-α binding with CBP/p300 and Sp1 in the nucleus of CAD cells. **(A)** Immunofluorescence imaging of ER-α’s interaction with CBP/p300 was detected in CAD cells after treatment with TX for 24 h. Scale bar, 20 μM. **(B)** Nuclear extracts from CAD cells treated with TX at several time points were immunoprecipitated with ER-α antibody, followed by Western blotting to detect CBP/p300 and Sp1 (*n* = 3). The data shown are representative of three independent experiments.

Since the nuclear ER-α was primarily involved in TX’s effects on the transcriptions of the ER’s target genes, we tested if TX recruited epigenetic coactivators, particularly histone acetyltransferases (HATs), CREB-binding protein (CBP)/p300, to interact with ER-α, as previously demonstrated in HeLa cells (Kraus and Kadonaga, 1998). The ER collaborates with a coactivator, CBP, to regulate the target gene expression, which facilitates ER binding to DNA and enhances the transcription of target genes (Waddell et al., 2021). CBP and p300 are highly homologous proteins that function as HATs and play key roles in transcriptional regulation. CBP/p300 was recruited to E2-responsive genes and enhanced transcriptional activity in MCF-7 cells (Yi et al., 2017). To determine whether TX facilitates this interaction, we performed ICC and co-IP assays. ICC imaging data confirmed the colocalization of ER-*α* and CBP/p300 within the nucleus (Figure 5A). The co-IP results showed that TX increased the interaction between ER-α and CBP/p300, as well as with ER-α and TF Sp1, which is known to interact with the ER-*α*/half-site ERE (Petz and Nardulli, 2000), in the nucleus of CAD cells (Figure 5B).

**Figure 6:**
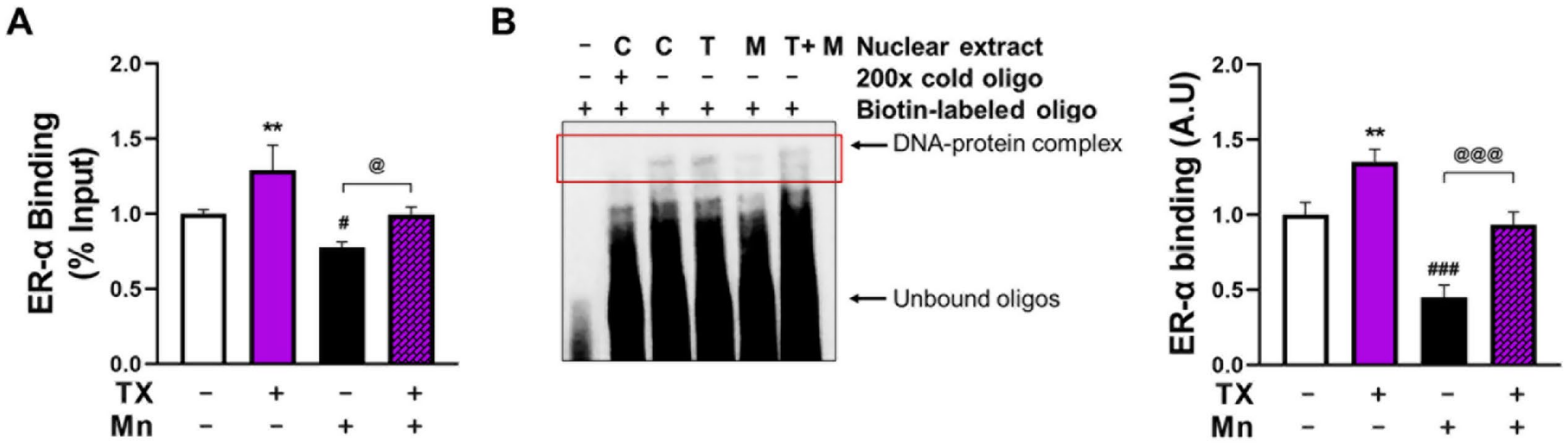
TX attenuated the Mn-induced reduction of ER-α binding to the ERE in the REST promoter. **(A)** After CAD cells were pre-treated with TX for 12 h, followed by Mn exposure for 24 h (in the presence of TX), a ChIP assay was performed to determine the binding of ER-α on the half-site ERE within the REST promoter *in vivo*, followed by quantification of ER-α-bound DNA by real-time qPCR. **(B)** EMSA was performed in nuclear extracts prepared from CAD cells treated with TX for 12 h, followed by Mn exposure for 24 h (in the presence of TX) as described in the Methods section. The upper black arrow shows the DNA–protein complex. **p < 0.01, ^#^*p* < 0.05, and ^###^*p* < 0.001, compared to the control. ^@^*p* < 0.05 and ^@@@^*p* < 0.001 compared to each other. (One-way ANOVA followed by *Sidak’s post-hoc*; *n* = 3). The data shown are representative of three independent experiments.

Next, we determined if TX and/or Mn modulates the binding of ER-α to the half-site ERE in the REST promoter by conducting DNA-protein interaction assays using ChIP and EMSA. The results revealed that TX increased the binding of ER-*α* to the ERE in the REST promoter and attenuated Mn-decreased ER-α binding to the ERE in the *in vivo* ChIP assay (Figure 6A), as well as the *in vitro* EMSA assay (Figure 6B).

### 3.4 Mutations on the half-site ERE motif in the REST promoter decreased REST promoter activity and reduced TX-induced REST promoter activity

**Figure 7:**
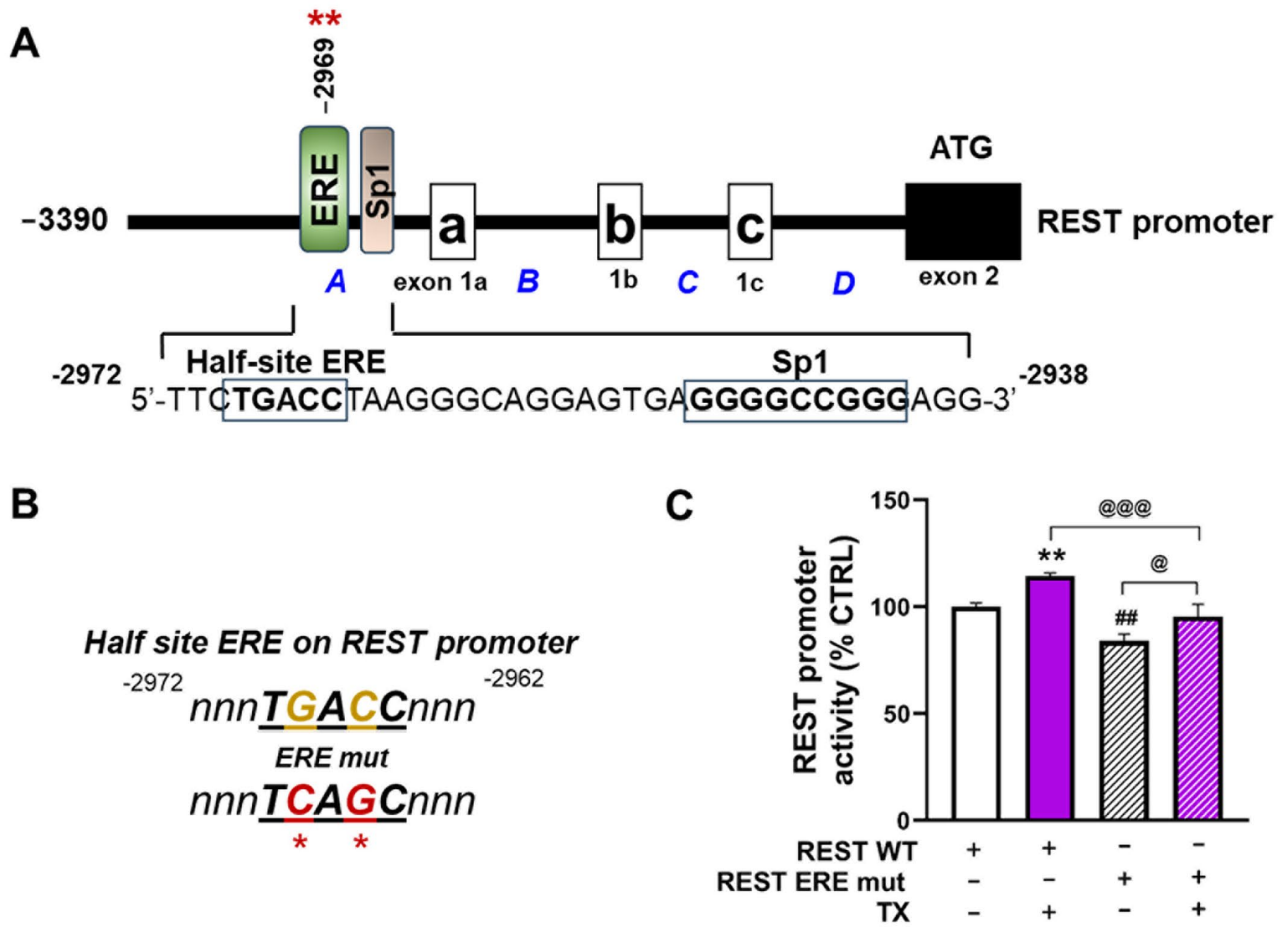
Mutations on the half-site ERE motif in the REST promoter decreased REST promoter activity and attenuated TX-induced REST promoter activity. **(A)** Illustration of the human 5′UTR REST promoter region with a half-site ERE and adjacent Sp1 binding motifs. **(B)** Two site-mutations of the half-site ERE consensus sequences on the human REST promoter plasmid are indicated in *red*. **(C)** CAD cells were transfected overnight with wild-type human 5′UTR REST promoter (REST WT) or ERE mutants of the REST promoter (REST ERE mut), then subsequently treated with TX for 6 h, followed by luciferase assay to detect REST promoter activity. **p < 0.01 and ^##^*p* < 0.01, compared to the control. ^@^*p* < 0.05 and ^@@@^*p* < 0.001, compared to each other. (One-way ANOVA followed by *Sidak’s post-hoc*; *n* = 3). The data shown are representative of three independent experiments.

To further confirm the critical role of ERE motifs of the REST promoter in TX-induced REST transcription, the ERE motifs were mutated by site-directed mutagenesis. The REST promoter-containing half-site ERE sequences *T****G****A****C****C,* were conserved in both human and mouse (Zhang et al., 2011). The ERE sequences were mutated to *T****C****A****G****C* in the human REST promoter plasmid construct, followed by promoter activity assay (Figures 7A,B). The results showed that mutation of the ERE significantly reduced REST promoter activity compared to the WT control, as well as attenuated TX-induced increase in REST promoter activity (Figure 7C), indicating that this ERE is critically involved in TX’s activation of the genomic ER in REST transcription.

## 4 Discussion

**Figure 8:**
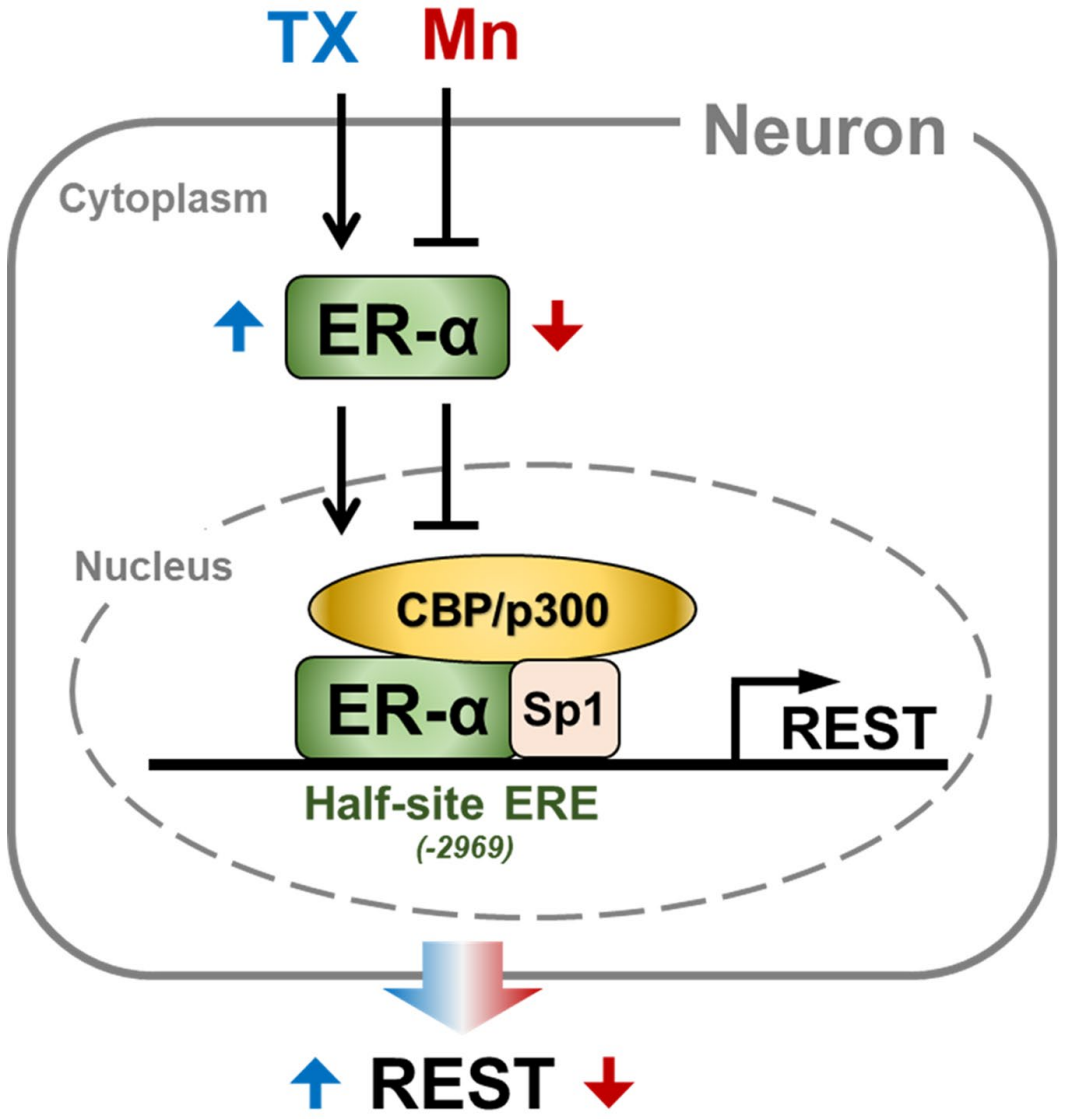
A schematic diagram of TX- and Mn-induced modulation of genomic ER-α, leading to REST expression. TX upregulates ER-α expression, promoting its translocation to the nucleus. The nuclear ER-α interacts with coactivators CBP/p300 and Sp1, enhancing its binding to the half-site ERE on the REST promoter. This interaction increases REST transcription in CAD neuronal cells. Conversely, Mn downregulates ER-α expression, reducing its nuclear translocation, resulting in decreased REST expression. Blue and red colors indicate TX- and Mn-induced effects, respectively.

The findings from the present study demonstrate that in CAD neuronal cells, TX upregulates REST transcription via the genomic ER-*α* mechanism. Mn inhibited this pathway, leading to the repression of REST transcription as well as reduction of ER-α protein levels, while TX mitigated these Mn effects in CAD neuronal cells. These findings were further supported by the results showing that TX increased ER-*α* protein levels, promoted its nuclear translocation, and enhanced its binding to the half-site ERE motifs in the REST promoter, in coordination with CBP and Sp1 (Figure 8). Since TX affords neuroprotection in several neurodegenerative diseases, such as PD, AD, as well as Mn neurotoxicity, our novel findings contribute to the understanding of the neuroprotective mechanisms of SERMs, which may lead to the development of neuro-SERMs, targeting the ER–*α*/REST pathway.

REST transcription is known to be regulated by several TFs, including Sp1 (Ravache et al., 2010) and TCF/LEF (Digman et al., 2025), but the involvement of ERE within the REST promoter was identified here for the first time. ER-*α* can act as a TF either by directly binding to a canonical palindromic ERE motif or by indirectly binding DNA via interaction with another TF in a tethered mode (Joseph et al., 2010). Although the full ERE (5′-GGTCAnnnTGACC-3′) consists of palindromic sequences (Bourdeau et al., 2004) typically required for ER binding for transcription of target genes, the half-site ERE can also contribute to gene regulation (Joseph et al., 2010). Our findings reveal that ER-*α* directly binds to the half-site ERE located in the exon 1A region of the human REST promoter, contributing to REST transcription. However, ER-*α* binding to this half-site ERE may require other factors, such as Sp1, to activate target gene expression (Petz and Nardulli, 2000). Sp1 enhances REST expression via its binding to the cis-elements in the REST promoter (Ravache et al., 2010). Notably, the half-site ERE is flanked by a Sp1 binding site, suggesting that Sp1 may facilitate ER-*α* recruitment or stabilization at this half-site ERE. In agreement with these results, TX promoted nuclear interaction between ER-*α* and Sp1, supporting a cooperative mechanism involving ER-*α*, the half-site ERE, and Sp1 in activating REST transcription. This aligns with previous reports showing that ER-α effectively binds to the half-site ERE when stabilized by its interactions with Sp1 bound to its nearby *cis*-elements (Klinge, 2001), as seen in the promoters of hsp27 (Porter et al., 1997) and TGF*α* (Vyhlidal et al., 2000).

The second ERE half-site (TGACC) appears to play a more critical role in target gene expression than the first half-site, as shown in the c-jun promoter, where it facilitates c-jun transcription via ER-α binding (Hyder et al., 1995), corroborating our data that the same sequences of TGACC half-site ERE were located in the REST promoter. Since mutating these ERE motifs in the REST promoter does not completely block the TX-induced REST activity, other non-genomic mechanisms likely contribute to this activation (Figure 7C). In fact, TX also activated the non-genomic ER-*α*/Wnt/*β*-catenin signaling to increase REST expression in CAD neuronal cultures (Digman et al., 2025), suggesting that TX upregulates REST via genomic, in addition to the non-genomic ER-α pathways.

As a SERM, TX induces ER-α agonistic effects in the brain in several experimental models (Zou et al., 2015; Lim et al., 2018). In this study, we showed that TX not only acted as an ER-α agonist, but also upregulated ER-α protein expression (Figure 3). Previous studies have shown that E2 could induce ER-α transcription in endothelial cells (Ihionkhan et al., 2002), suggesting a potential positive feedback mechanism, which may explain TX-induced increases in ER-*α* protein expression in the present study. Further studies are needed to elucidate how TX increases ER-α expression. Furthermore, the present study demonstrated that activation of ER-β suppresses the ER-α–REST pathway (Supplementary Figures S2, S3), consistent with previous reports indicating that ER-β functions as a negative regulator of ER-α (Hall and McDonnell, 1999). This finding highlights a complex regulatory interplay, as TX has been shown to activate both ER-α and ER-β (Gruvberger-Saal et al., 2007). It is plausible that the TX-induced increase in ER-α expression outperforms the suppressive effects of ER-β on the ER-α–REST axis. However, further studies are required to understand TX-induced inter-regulation of ER-α and ER-β.

Mn decreased ER-α protein levels in CAD neuronal cultures (Figure 4), corroborating findings in mice (Pajarillo et al., 2018), suggesting that the downregulation of ER-α represents a potential mechanism by which Mn induces its neurotoxicity. Dysregulation of ER-α expression appears to be common for neurotoxins, since studies have shown that the PD neurotoxin 1-methyl-4-phenyl-1,2,3,6-tetrahydropyridine (MPTP) decreased ER-α levels in mice (D'Astous et al., 2004) and paraquat, another PD-modelling neurotoxin, also reduced ER-α levels in PC12 cells (Gelinas et al., 2004).

Activation of the genomic ER pathway has been identified as a critical mechanism underlying the neuroprotective effects of several SERMs. Bazedoxifene has been shown to improve cognitive deficits in ovariectomized mice via genomic ER signaling in the brain (Hill et al., 2020), corroborating our finding that TX enhances REST transcription via the genomic ER mechanism. Similarly, raloxifene protects against Mn-induced glutamate excitotoxicity via ER-α (Karki et al., 2014), and ospemifene attenuates hypoxia- and ischemia-induced neuronal apoptosis by ER-α (Pietrzak et al., 2023).

Although the current study using TX showed potential for the development of neuro-SERMs that activate the ERs only in the brain, there is a limitation to the TX-REST axis as a neuroprotection mechanism because of the lack of *in vivo* and functional studies. A future study is required to determine TX’s neuroprotection in the absence of REST in the brain. Notably, global REST-deleted mice are embryonic lethal (Chen et al., 1998), and neuronal REST deletion in the brain exacerbates a PD-toxin MPTP toxicity (Huang et al., 2019), indicating that REST is protective in the brain. Additionally, we have recently found that dopaminergic REST plays a role in TX’s protective effects against Mn toxicity in mice (manuscript under review), although other mechanisms are also involved in TX effects.

In conclusion, our findings demonstrate that TX enhances REST expression by activating genomic ER-α signaling, thereby counteracting Mn-induced suppression of this pathway. Considering the established neuroprotective role of REST in multiple neurodegenerative disorders and E2’s unwanted peripheral effects, targeting the genomic ER-α–REST pathway by the development of brain-selective SERMs (neuroSERMs) (Zhao et al., 2005) presents a promising therapeutic strategy.

## Data Availability

The original contributions presented in the study are included in the article/Supplementary material, further inquiries can be directed to the corresponding author.
