# Supplementary material for: Tamoxifen attenuates manganese-induced dysregulation of neuronal REST via the genomic ER-α mechanism
Source: Front Mol Neurosci. 2025 Sep 15;18:1648904. doi: 10.3389/fnmol.2025.1648904 (PMC12477136; doi:10.3389/fnmol.2025.1648904)
Supplement: Supplementary file 1 [file Data_Sheet_1.PDF]

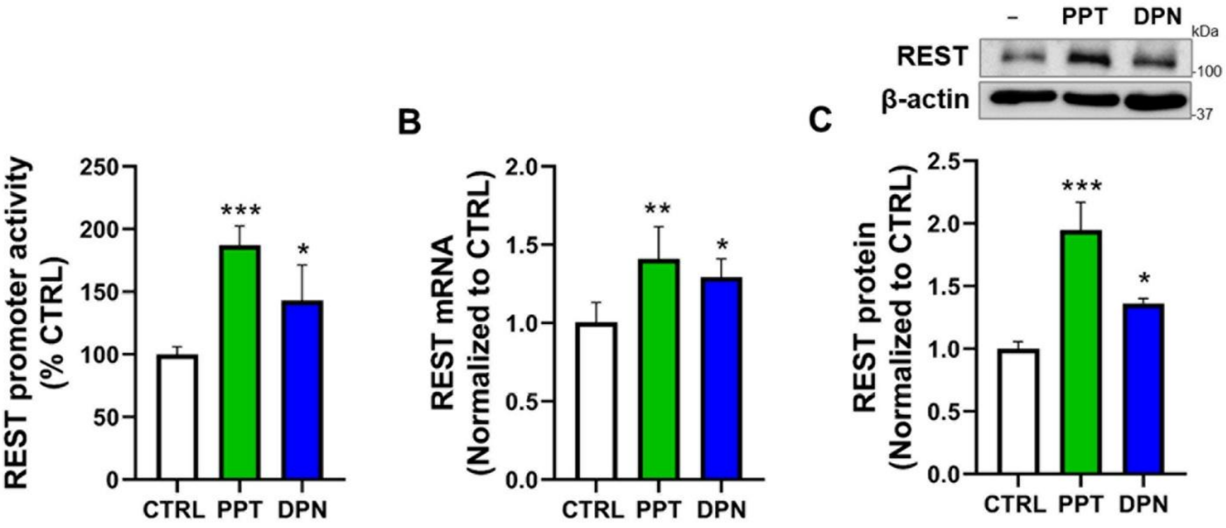

Supplementary Figure 1. ER-α agonist PPT (100 nM) and ER-β agonist DPN (100 nM) increased REST expression in CAD cells. **(A)** CAD cells were transfected with a human 5'UTR-REST promoter vector and then exposed to either 100 nM PPT or 100 nM DPN for 6 h, followed by a luciferase assay to measure REST promoter activity. **(B, C)** CAD cells were treated with either 100 nM PPT or 100 nM DPN for 12 h and 24 h, followed by measurement of REST mRNA using qRT-PCR **(B)** and REST protein using western blot **(C)**, respectively. GAPDH and β-actin were used as loading controls for mRNA and protein, respectively. Quantification of protein bands was normalized to β-actin. Relative mRNA expression levels were normalized to GAPDH. *$p<0.05$, **$p<0.01$, ***$p<0.001$, compared to control. (One-way ANOVA followed by *Sidak's* post hoc, n=3).

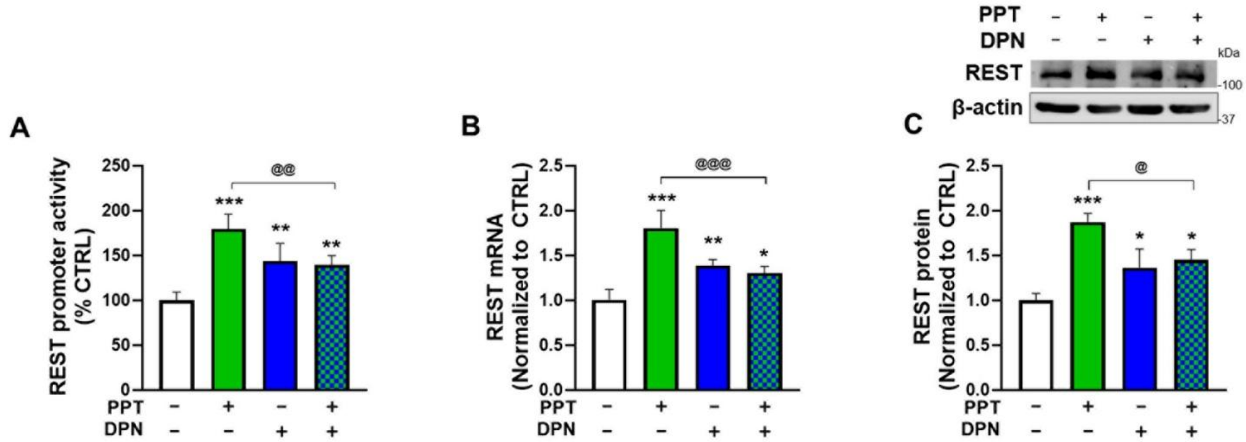

Supplementary Figure 2. ER-β activation blocked the increasing effects of ER-α on REST expression in CAD cells. **(A)** CAD cells were transfected with a human 5'UTR-REST promoter vector, then co-treated with 100 nM PPT and 100 nM DPN for 6 h, followed by measurement of REST promoter activity. **(B, C)** CAD cells were co-treated with 100 nM PPT and 100 nM DPN for 12 and 24 h, followed by measurement of REST mRNA **(B)** and protein **(C)**, respectively. GAPDH and β-actin were used as loading controls for mRNA and protein, respectively. Quantification of protein bands was normalized to β-actin. Relative mRNA expression levels were normalized to GAPDH. *p<0.05, **p<0.01, ***p<0.001, compared to control. $^{@}$p<0.05, $^{@@}$p<0.01, $^{@@@}$p<0.001, compared to each other. (One-way ANOVA followed by *Sidak's* post hoc; n=3). The data shown are representative of 3 independent experiments.

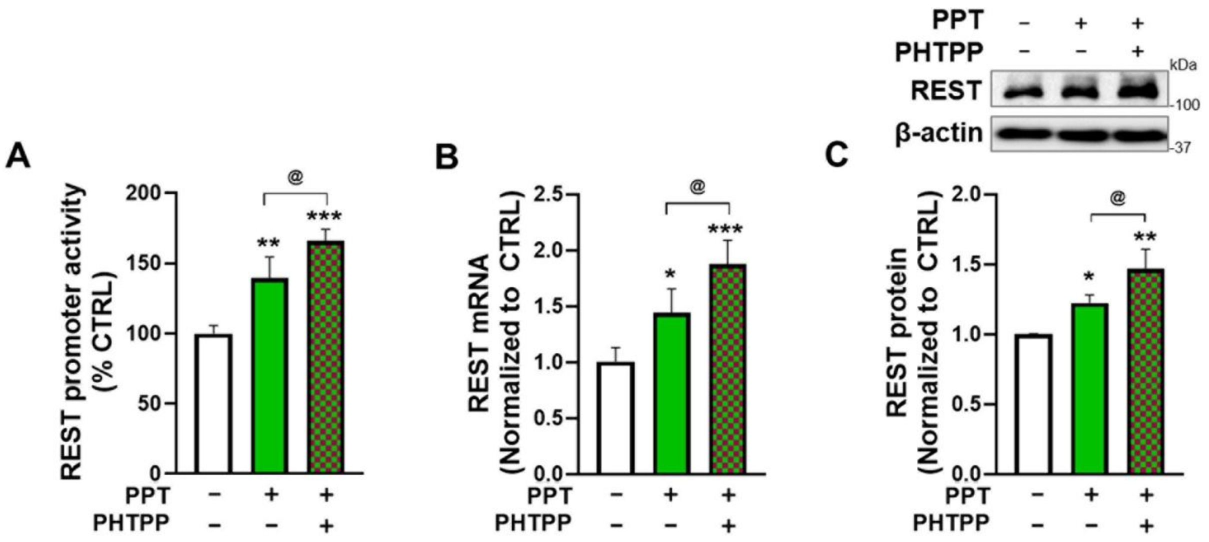

Supplementary Figure 3. Blocking ER-β further increased ER-α-induced REST expression in CAD cells. **(A)** CAD cells were transfected with a human 5'UTR-REST promoter vector, then treated with 100 nM PPT or with 100 nM PHTPP (ER-β antagonist) for 6 h, followed by measurement of REST promoter activity. **(B, C)** CAD cells were treated with 100 nM PPT or with 100 nM PHTPP for 12 h for REST mRNA **(B)** and 24 h for REST protein **(C)**, respectively. GAPDH and β-actin were used as loading controls for mRNA and protein, respectively. Quantification of protein bands was normalized to β-actin. Relative mRNA expression levels were normalized to GAPDH. *$p<0.05$, **$p<0.01$, ***$p<0.001$, compared to control. $^{@}p<0.05$, compared to each other. (One-way ANOVA followed by *Sidak's* post hoc; n=3).
